# 3D bioprinted ferret mesenchymal stem cell-laden cartilage grafts for laryngotracheal reconstruction in a ferret surgical model†

**DOI:** 10.1039/d4bm01251h

**Published:** 2025-01-22

**Authors:** Alexandra McMillan, Matthew R. Hoffman, Yan Xu, Zongliang Wu, Emma Thayer, Adreann Peel, Allan Guymon, Sohit Kanotra, Aliasger K. Salem

**Affiliations:** a Department of Otolaryngology, University of Iowa Hospitals and Clinics Iowa City IA USA; b Department of Pharmaceutical Sciences and Experimental Therapeutics, College of Pharmacy, University of Iowa Iowa City IA USA aliasger-salem@uiowa.edu; c Department of Chemical and Biochemical Engineering, University of Iowa Iowa City IA USA; d Department of Head and Neck Surgery, UCLA Los Angeles California USA

## Abstract

Chondrogenic differentiation of mesenchymal stem cells (MSCs) within a three-dimensional (3D) environment can be guided to form cartilage-like tissue *in vitro* to generate cartilage grafts for implantation. 3D bioprinted, MSC-populated cartilage grafts have the potential to replace autologous cartilage in reconstructive airway surgery. Here, bone marrow-derived ferret MSCs (fMSCs) capable of directed musculoskeletal differentiation were generated for the first time. A multi-material, 3D bioprinted fMSC-laden scaffold was then engineered that was capable of *in vitro* cartilage regeneration, as evidenced by glycosaminoglycan (GAG) production and collagen II immunohistochemical staining. *In vivo* implantation of these 3D bioprinted scaffolds in a ferret model of laryngotracheal reconstruction (LTR) demonstrated healing of the defect site, epithelial mucosalization of the inner lumen, and expansion of the airway volume. While the implanted scaffold allowed for reconstruction of the created airway defect, minimal chondrocytes were identified at the implant site. Nevertheless, we have established the ferret as a biomedical research model for airway reconstruction and, although further evaluation is warranted, the generation of fMSCs provides an opportunity for realizing the potential for 3D bioprinted regenerative stem cell platforms in the ferret.

## Abstract 2

Chondrogenic differentiation of mesenchymal stem cells (MSCs) within a three-dimensional (3D) environment can be guided to form cartilage-like tissue *in vitro* to generate cartilage grafts for implantation.

## Graphical abstract

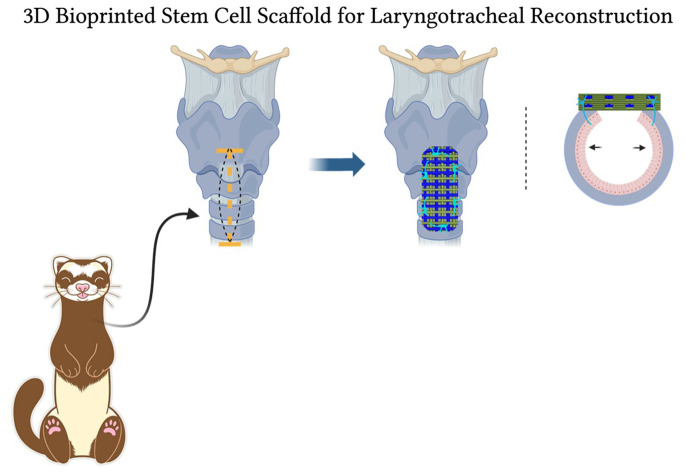

## Introduction

Congenital or acquired laryngotracheal stenosis (LTS) in the pediatric population is a narrowing of the upper airway that can result in significant upper airway obstruction.^1,2^ An incidence of LTS as high as 44% has been cited after prolonged or repeated endotracheal intubation in low-birthweight neonates.^3^ The goal of LTS management is to maintain a prosthetic-free, patent airway. The mainstay treatment for higher grade stenosis and patients who have failed more conservative approaches, including endoscopic and medical treatment, is laryngotracheal reconstruction (LTR). LTR is a surgical approach that addresses the narrowed airway by opening and expanding it with a cartilage graft placed anteriorly or anteriorly/posteriorly at the level of the stenosis.^4^ Current autogenic graft options employed often include thyroid alar cartilage, auricular cartilage, or, most commonly, costal cartilage from the rib.^5^ Despite excellent surgical results, cartilage graft harvest is associated with disadvantages such as donor site morbidity, additional surgical time, limited tissue availability, and surgical complications such as pneumothorax in the case of rib cartilage harvest. Alternatives including the use of irradiated allogenic costal cartilage^6^ and alcohol-stored allogenic auricular cartilage^7^ have been investigated in animal models; however, both approaches demonstrate resorption. Tissue engineering (TE) offers an alternative to native cartilage for airway surgery. Studies pertaining to tracheal engineering have demonstrated application in patch tracheoplasty^8,9^ as well as full circumferential tracheal implantation for application in tracheal resection, the latter of which typically involves engineering rigid polymeric tubes.^10,11^ Although TE grafts have been engineered for application in airway reconstruction,^12^ few studies have focused on application in LTR.

One approach that has emerged as a powerful tool to advance the field of regenerative medicine and has great potential to engineer cartilage for airway surgery is 3D printing. This technique allows for fabrication of individualized constructs tailored to the desired physiological and biomechanical properties of the implant by controlling shape and deposition of print material. Only one report has employed 3D printing for application in LTR surgery.^13^ Constructs were 3D printed using melt extrusion of resorbable thermoplastic polymer alone followed by manual coating of the matrix with a cell-laden hydrogel.^14^ As opposed to previously reported conventional 3D printing,^11,14–16^ 3D bioprinting allows for direct printing of a bioink composed of live cellular material, typically in combination with a biocompatible, biodegradable hydrogel. This offers the advantage of control over spatial distribution of the bioink (*i.e.*, control over cell distribution)^13,17^ which can allow for creation of individualized and complex constructs.

In addition to graft design, animal models are indispensable for developing implantable therapies for LTS. Reports employing TE grafts for LTR have utilized rabbit^14,18,19^ and porcine^3^ animal models. Alternatively, the domestic ferret (*Mustela putorius furo*) may serve as an attractive animal LTR model. Ferrets have proven an excellent species for modeling airway pathophysiology and have expanded human disease research related to cystic fibrosis,^20^ respiratory viral studies,^21^ and subglottic stenosis.^22,23^ This is due to similar physiology to that of humans and a relatively large airway to body size.^24^ Ferrets, like humans, have submucosal glands throughout their proximal airway unlike mice, which secrete a more watery surfactant,^24^ and rabbits, which lack these submucosal glands.^25^ Rodent and rabbit models provide limited evaluation of cell therapy strategies due to these differences in physiology as well as small size.^24^ Although pigs have been used to mimic human airway pathology, ferrets have the potential to serve as a valuable preclinical model for airway surgery including LTR due to ease of use compared to pigs.^26^

Despite the benefit of ferrets as a preclinical airway model, no studies, to date, have applied TE to study regeneration in ferrets. Additionally, ferret mesenchymal stem cells (fMSCs) have yet to be described in the literature. Bone-marrow derived MSCs are popular for application in musculoskeletal regeneration as they are readily available from bone marrow, undergo self-renewal, and have the capacity to differentiate down chondrogenic, osteogenic, and adipogenic lineages.^27^ MSCs may be ideal for transplantation due to their low immunogenicity, non-tumorigenic characteristics, and immunomodulatory properties, distinct from their capacity for differentiation.^28,29^ Despite known challenges of using MSCs in clinical applications for cartilage regeneration,^30,31^ their use has been applied to various new approaches in regenerative medicine, including 3D printing, with great promise.^13,32,33^ This study aims to generate fMSCs capable of directed musculoskeletal differentiation to provide the first step towards surgical implantation of MSC-populated grafts in a ferret without the use of immunosuppression. 3D bioprinted scaffolds composed of fMSC-laden gelatin methacrylate (GelMa) bioink co-printed with polycaprolactone (PCL) were developed to fabricate multi-material cartilage grafts which were then characterized following *in vitro* culture and *in vivo* implantation in a ferret model of LTR. This is a first step towards evaluating MSC-based regenerative therapies in the ferret.

## Materials and methods

### fMSC isolation and culture

Bone marrow-derived fMSCs were obtained from three ferret donors. Wild-type (WT), domestic female ferrets (*Mustela putorius furo*) ranging from 2–12 months old were used for fMSC harvest. Female ferrets were used due to ease of housing. Immediately after euthanasia, fMSCs were isolated from femoral bone marrow *via* density gradient centrifugation and differential cell adhesion to tissue culture plastic based on modification of a previously described protocol (ESI Fig. 1†).^34^ Briefly, bone marrow was harvested from the ferret femora and collected into a sterile 50 ml conical tube containing 20–30 ml of Dulbecco's Modified Eagle's Medium-High Glucose (DMEM-HG, Gibco, Billings, MT, USA). The mixture was centrifuged at 450*g* for 5 min, the supernatant was discarded, and the remaining precipitate was resuspended with 4 ml DMEM-HG. The cell suspension was carefully layered onto 3 ml of Ficoll-Paque® (GE Health Care Biosciences, Piscataway, NJ, USA) in a 15 ml-conical tube and centrifuged at 400*g* for 40 minutes. After centrifugation, the interphase was harvested and mixed with at least three volumes of DMEM-HG to wash the cells and centrifuged at 450*g* for 10 min and the supernatant was removed. The cell pellet was mixed with growth medium composed of DMEM-HG supplemented with 10% fetal bovine serum (FBS; R&D Systems, Minneapolis, MN, USA) and 1% penicillin–streptomycin (1000 units per ml–1000 μg ml^−1^, respectively; P/S, Gibco). The culture medium was carefully changed after 3 days and every 3–4 days thereafter. Beyond passage 1, medium was supplemented with 10 ng ml^−1^ fibroblast growth factor-2 (FGF-2, R&D Systems). Passage 4 fMSCs were used for all experiments. Cells were maintained at 37 °C with 5% CO_2_ in a humidified incubator.

### Colony forming unit assay

The colony forming unit (CFU) potential of the primary nucleated cell population of isolated cells was determined by culturing fMSCs from donors 1, 2, and 3 expanded without FGF-2 supplementation in triplicate into 6 well plates cultured with 2 ml of growth medium. Cells were plated at 50 and 100 cells per cm^2^ for 7 days. Cells were fixed with 10% neutral buffered formalin (NBF) and stained with 0.01% (w/v) crystal violet solution for 15 min, washed, and photographed. Acetic acid elution of crystal violet was used to semi-quantify CFU and the absorbance measured at 590 nm (SpectraMax microplate reader, Molecular Devices LLC, Sunnyvale, CA).^35^

### 3D fMSC aggregate formation and musculoskeletal differentiation

The base medium (basal pellet medium (BPM)) for chondrogenic and osteogenic medium for 3D cell aggregate differentiation consisted of DMEM-HG supplemented with 1% ITS^+^ Premix (Corning Inc., Corning, NY, USA), 1% sodium pyruvate (Gibco), 1% nonessential amino acids (NEAA; Lonza Group, Basel, Switzerland), and 1% P/S. Chondrogenic medium consisted of BPM supplemented with 100 nM dexamethasone (Sigma Aldrich, St Louis, MO, USA) and 37.5 mg ml^−1^l-ascorbic acid-2-phosphate (Wako Chemicals USA Inc., Richmond, VA, USA). Osteogenic medium consisted of BPM supplemented with 1 nM dexamethasone, 10 mM β-glycerophosphate (EMD Millipore, Burlington, MA, USA), and 50 mg ml^−1^l-ascorbic acid-2-phosphate. FMSC aggregates were formed in a manner similar to that previously described.^34,36^ Briefly, cells from three donors were trypsinized and suspended at a concentration of 1.25 × 10^6^ cells per ml in either chondrogenic or osteogenic medium, and a 200 μl cell suspension solution was dispensed per well into sterile 96-well V-bottom polypropylene microplates. The plates were centrifuged at 500*g* for 5 min to form aggregates, and the medium was changed every other day. Aggregates were formed and cultured with and without growth factor (GF) supplementation. For chondrogenic differentiation, chondrogenic medium was supplemented with 10 ng ml^−1^ TGFβ-1 (PeproTech, Rocky Hill, NJ, USA) for three weeks. Aggregates for osteogenic differentiation were formed and cultured in osteogenic medium supplemented with 100 ng ml^−1^ BMP-2 for three or five weeks. GF-free cultures served as controls. Medium was changed every 2 days. Aggregates were harvested and stored at −20 °C prior to biochemical analysis (*N* = 3 aggregates per condition, per time point), or fixed immediately in 10% NBF prior to histology (*N* = 3 aggregates per condition), as described below.

### Quantitative biochemical analysis of fMSC aggregates

Chondrogenic differentiation of aggregates designated for biochemical analysis were analyzed at 3 weeks for glycosaminoglycan (GAG) and DNA quantification (*N* = 3 aggregates per condition) according to a previously reported protocol.^37^ Briefly, aggregates were digested overnight in 1 ml papain buffer at 65 °C. GAG was measured with dimethyl-methylene blue (DMMB, Sigma-Aldrich) at an absorbance of 595 nm, and DNA analysis was performed with a Picogreen assay kit at fluorescence of 480/520 nm (Thermo Fisher, Waltham, MA).

Similarly, osteogenic differentiation of aggregates was analyzed at 3 and 5 weeks and assayed for DNA, calcium content, and alkaline phosphatase (ALP) activity (*N* = 3 aggregates per condition per time point) according to a previously reported protocol.^38^ Briefly, aggregates were homogenized (Tissue Tearor, BioSpec Products, Bartelsville, OK, USA) twice for 30 s on ice in 1 ml papain buffer. ALP assay buffer (Abcam, Cambridge, UK) was added 1 : 1 to a portion of each sample for ALP activity analysis. ALP activity was measured using an ALP assay kit (Sigma-Aldrich) according to the manufacturer's instructions and compared to a standard curve of *para*-nitrophenol (Sigma-Aldrich) after incubation with *para*-nitrophenylphosphate (pNPP, EMD Millipore) at 37 °C and read at 405 nm. ALP activity is expressed as amounts of converted substrate read from the *para*-nitrophenol standard curve (μM) relative to time of incubation. The remainder of each sample was digested overnight after mixing 1 : 1 with papain buffer at 65 °C. The next day, an aliquot of this sample was treated with 1 M HCl to dissolve calcium crystals and assayed for calcium content using an *o*-cresophthalein complexone assay (Pointe Scientific, Canton, MI, USA) and a standard curve and analyzed using a plate reader at 570 nm. The remainder of the aliquot was used to quantify DNA by mixing 1 : 1 with 10% EDTA in 0.05 M Tris-HCl buffer (pH7.4), and DNA analysis was performed as previously described.

### Histologic and immunohistochemical analysis of fMSC aggregates

Aggregates cultured in chondrogenic medium with and without exogenous TGFβ-1 were fixed overnight in 10% NBF, embedded in paraffin, sectioned into 5 μm slices, and mounted sections stained with Safranin-O (SafO) with Fast Green counterstain to identify the presence of sulfated GAGs (sGAGs), and hematoxylin and eosin (H&E) to examine chondrogenic morphology. The stained sections were examined using a light microscope (Olympus BX61 microscope; Olympus, Center Valley, PA, USA). Similarly, aggregates cultured in osteogenic medium with and without exogenous BMP-2 were harvested and processed in a similar fashion. Mounted tissue sections were stained with H&E and Alizarin Red S (ARS) stain (Sigma Aldrich), the latter of which stains calcium. Immunohistochemistry (IHC) was performed on aggregates to analyze the presence of collagen types I, II, or X (col I, II, X). Briefly, IHC was performed by deparaffinization and rehydration of tissue sections with decreasing concentrations of ethanol. Endogenous peroxidase activity was quenched per manufacturer's protocol (Abcam) for 10 min, and protease (1 mg ml^−1^; Sigma-Aldrich) was applied to each section for 30 min for epitope retrieval. Anti-col I (1 : 100, SAB4500362; Abcam), anti-col II (1 : 200, NBP1-77795; Novus biologics), and anti-col X (1 : 200, PA5-115039, Thermo Fisher) were used as primary antibodies and detected according to the manufacturer's instructions. Slides were counterstained with 0.05% Fast Green, dehydrated in increasing concentrations of ethanol, cover slip mounted with Permount (Thermo Fisher), and images acquired (*N* = 3 aggregates per condition). Ferret trachea and decalcified knee cartilage were harvested and processed in a similar manner to serve as positive controls for staining. Negative controls were used to validate antibody specificity using PBS in place of primary antibody.

### Osteogenic differentiation of monolayer fMSCs

To compare osteogenic differentiation of the three donor fMSCs in monolayer culture, fMSCs were plated at 30 000 cells per well in triplicate per condition in 24 well plates coated with fibronectin (2 μg per well; Sigma-Aldrich) per manufacturer's instructions. Cells were cultured in osteogenic monolayer medium composed of DMEM-low glucose (DMEM-LG, Gibco), 10% (v/v) FBS, 1% P/S, 100 nM dexamethasone, 10 mM β-glycerophosphate, 50 μM ascorbic acid, with and without supplementation of 100 ng ml^−1^ BMP-2 for 4 weeks with media change every 3–4 days. Cells were fixed for 30 min in 10% NBF, washed, stained with ARS for 15 min to assess calcium staining, washed, and photographed. ARS staining was then quantified with acetic acid elution against a standard curve and absorbance measured with a plate reader at 405 nm.^39,40^

### Bioink preparation and cell density characterization of 3D bioprinted discs

3D bioprinted cylindrical discs (8 mm diameter × 1 mm height; G-code graciously provided by Tomas Gonzalez Fernandez^41^) with varying cell concentrations (5 × 10^6^, 10 × 10^6^, 20 × 10^6^ cells per ml; 5E6, 10E6, 20E6, respectively) were prepared according to the manufacturer's instructions (Cellink, Gothenburg, Sweden) in BPM. Briefly, lyophilized GelMa with 50%-degree substitution was mixed with reconstitution buffer containing 0.25% lithium phenyl-2,4,6-trimethylbenzoylphosphinate (LAP) photoinitiator to produce a GelMa 10% (w/w) solution. The lyophilized GelMa was dissolved in the LAP solution under constant stirring on a heat plate at 50 °C. The gel-liquid phase of GelMa is temperature dependent with liquid phase achieved at higher temperatures, typically over 32 °C.^42^ Thus, GelMa solution was brought to 37 °C (liquid phase) and thoroughly mixed ten parts bioink with one part cell suspension. The prepared cell-GelMa solutions were loaded into the temperature-controlled, pneumatic printhead and maintained at 27 °C for printing. GelMa strands were printed using a 25G (0.437 mm inner diameter) Micron-S precision conical metal nozzle (Fisnar, Germantown, WI) at a deposition speed of 4 mm s^−1^ and pneumatic pressure ranging from 10–60 kPa (Table 1). The discs were then photocrosslinked *via* application of 405 nm wavelength light for 15 seconds at a distance (height) of 25 mm following printing of each condition (*n* = 4 discs). The 3D-bioprinted discs were transferred to 10 cm Petri dishes with 20 ml of growth medium overnight and then transferred to 6 well plates with 3 ml chondrogenic medium supplemented with TGFβ-1 (10 ng ml^−1^) for 21 days in an incubator at 37 °C and 5% CO_2_ with media change every 2 days. Hydrogels were harvested for biochemical analysis at day 21 post-print. Each disc was washed in PBS and frozen at −80 °C for biochemical analysis, as described above (*n* = 4 discs per condition). Donor 1 fMSCs were used. Cell-free GelMa bioink served as controls (Ctrl).

**Table 1:** Print parameters for 3D bioprinted scaffolds

	Print parameters		
Material	Temperature (°C)	Speed (mm s^−1^)	Pressure (kPa)
GelMa 10% w/w	27	4	10–90
PCL 25 kDa	85	5	150

### 3D bioprinted hybrid scaffold fabrication and chondrogenic differentiation

Multi-material, porous scaffolds were designed with overall dimensions measuring 20 mm (length) × 20 mm (width) × 1.44 mm (height). Structures were fabricated using the Cellink BioX printer to allow for co-printing strands of cell-free and cell-laden GelMa (20 × 10^6^ cells per ml; 20E6) bioink filaments alternating with PCL filaments (GelMa only/PCL and GelMa + cells/PCL, respectively). PCL strands were printed 2 mm apart and GelMa was printed between the two PCL strands. One layer of GelMa was printed for every two repeated layers of PCL except for the final top layer which was composed of a single layer of each. The number of repeated layers with consecutive paths printed in an alternating 0–90° perpendicular pattern (Fig. 3A, ESI Fig. 5†) resulted in 5 total layers.

For the fabrication, GelMa bioink was prepared and bioprinted as described above (Table 1). GelMa was photocrosslinked at 405 nm for 15 seconds at a distance (height) of 25 mm after each bioink layer. PCL (25 000 kDa, Polysciences, Warrington, PA, USA) pellets were loaded into the Cellink thermoplastic print head, heated up to 150 °C to prevent potential microbial contamination and then set to the dispensing temperature of 85 °C. 3D bioprinting was performed with melted PCL strands dispensed from a 300 μm inner diameter metal nozzle at a deposition speed of 5 mm s^−1^ and a pneumatic pressure of 150 kPa. The completed constructs take approximately 20 minutes to print. Cells were suspended in the GelMa solution for up to 4 hours. Printed constructs are then placed in a 10 mm Petri dish containing 20 ml of growth medium overnight in an incubator at 37 °C and 5% CO_2_. On day 1 post-print, the scaffolds were sectioned in half and one half utilized for live/dead and DNA analysis, as described below. The second half of the scaffold was subsequently transferred into 6 well plates with 3 ml of chondrogenic medium with TGFβ-1 supplementation (10 ng ml^−1^) and cultured for 21 days. The culture medium was replaced every 2 days. At the endpoint, scaffolds were harvested for biochemical analysis and histology. The same computer assisted design (CAD) design minus the PCL filaments was used to print GelMa only scaffolds both without (GelMa only) and with 20E6 encapsulated cells (GelMa + cells) for comparison. The scaffolds were prepared with donor 1 fMSCs.

#### Biochemical analysis

Biochemical analysis of the scaffolds was evaluated at day 1 and 21 post-print. The portion of each scaffold designated for biochemical analysis was washed in PBS and stored at −80 °C prior to analysis. Punch biopsies of scaffolds were obtained and subsequently lyophilized. GAG and DNA analysis was performed as described above and normalized to area of the sample obtained from the punch biopsy. Scaffolds were printed in triplicate during three separate printing sessions for biochemical analysis.

#### Histologic and immunohistochemical analysis

Chondrogenesis of 3D bioprinted scaffolds was assessed with IHC and histological analysis after 21 days of culture. The section of each scaffold designated for IHC analysis was harvested and assessed using col II staining, phalloidin, and DAPI. Scaffolds were harvested and fixed in 4% PFA for 30 min. The scaffolds were permeabilized using 0.5% Triton X in PBS, washed 3× in PBS, and blocked with 3% bovine serum albumin (Sigma). Next, sections were incubated with the primary antibodies for col II and phalloidin (Alexa Fluor 546 phalloidin, Invitrogen) for 1 hour at room temperature (RT). Subsequently, sections were incubated with the secondary antibody Alexa Fluor 488 (1 : 1000; donkey anti-rabbit IgG; Invitrogen, Waltham, MA) at RT for 1 h. Finally, sections were cover-slip mounted with DAPI mounting medium. Composite *z*-stack images (60 μm) of the scaffolds were obtained by a confocal laser scanning microscope (Leica SP8 confocal, Wetzlar, Germany). Scaffolds were printed in triplicate during three separate printing sessions for IHC analysis. Negative controls were used to validate antibody specificity using PBS in place of primary antibody.

A section of each scaffold was harvested for paraffin-embedded histological analysis of the neo-tissue after 21 days of *in vitro* culture. The scaffolds were harvested and fixed in 10% NBF overnight, washed three times with PBS, and embedded in low melt paraffin (42–44 °C, EMD Millipore, Burlington, MA, USA). Samples were sectioned into 5 μm slices and mounted sections stained with SaFO with Fast Green counterstain to identify the presence of sGAGs, Masson Trichrome stain to identify the presence of collagen, and hematoxylin stain to further identify chondrocyte morphology. The stained sections were examined using a light microscope. Scaffolds were printed in triplicate.

#### Live/dead analysis

Cell viability was assessed at day 1 post-print by live/dead assay staining kit according to the manufacturer's instructions (Biotium, San Francisco, CA, USA). The live cells were dyed green with calcein and the dead cells were dyed red with ethidium bromide. Composite *z*-stack images (80 μm) of the scaffolds were obtained by a confocal laser scanning microscope. The number of live and dead cells was quantified with compressed images using Image J software analysis (National Institutes of Health, Bethesda, Maryland, USA). Scaffolds were printed in triplicate during three separate printing sessions for cell viability analysis.

#### Mechanical testing

Scaffolds were 3D bioprinted, sectioned in half, and cultured in 6 well plates with 3 ml of chondrogenic medium supplemented with TGFβ-1 for 21 days, as described above. Scaffolds were harvested, washed with PBS, and stored at −80 °C prior to mechanical testing. Samples were then thawed in PBS and stored on ice until mechanical testing was performed on 8 mm punch biopsies. Unconfined compression testing with a 90 N force applied at a rate of 0.5 mm min^−1^ was performed using a Admet eXpert 7601 tension testing system (Norwood, MA, USA) and Young's modulus was calculated, which is an objective measurement of stiffness of a material and is defined as the compressive stress divided by the compressive strain.^43^ It is used here to measure the biomechanical integrity of each of the grafts. The mean data from at least *n* = 2 punches per scaffold, *n* = 3 scaffolds per condition is presented.

### *In vivo* graft implantation

All animal procedures were performed in accordance with the Guidelines for Care and Use of Laboratory Animals at the University of Iowa and approved by the University of Iowa Institutional Animal Care and Use Committee. The University of Iowa is accredited by AAALACi (#000833) and has PHS Animal Welfare Assurance (D16-00009, A3021-01).

#### Subcutaneous implantation

3D bioprinted GelMa + cells/PCL scaffolds were implanted (*n* = 4) subcutaneously into the back of an adult female WT domestic ferret with one scaffold inserted into separate pockets following sedation as described below in order to evaluate the role of site location implantation on the constructs given the increased vascularity of subcutaneous implantation compared to the trachea. The constructs were harvested after 5 weeks for paraffin-embedded histology, as described above.

#### Laryngotracheal reconstruction

This study was approved by the institutional animal care and use committee at the University of Iowa (Table 2). To evaluate the *in vivo* efficacy of cartilage regeneration of the engineered graft in an anterior graft LTR model, surgery was performed in *n* = 4 adult female WT domestic ferrets with implantation of a 3D bioprinted GelMa + cells/PCL scaffold (20 × 10^6^ cells per ml) by a board-certified laryngologist/pediatric otolaryngologist (MRH). Prior to implantation, scaffolds were sectioned in half and cultured in 6 well plates with 3 ml of chondrogenic medium supplemented with TGFβ-1 for 21 days, as previously described. Ferrets weighing > 700 grams were used for these studies. Preoperatively, ferrets were administered ketamine (6 mg kg^−1^)/xylazine (1 mg kg^−1^), subcutaneously for sedation. Subcutaneous buprenorphine (0.08 mg kg^−1^) provided analgesia prior to surgery. Bupivacaine was administered locally to the vertical midline incision of the neck. Dexamethasone (1 mg kg^−1^) and Baytril (10 mg kg^−1^) were administered as prophylaxis for airway edema and infection,^44^ respectively, and for three days post-operation. Ferrets were induced and maintained with isoflurane (1%–5%) with oxygen (100%) administered by mask followed by intubation with a 2.5 mm cuffed endotracheal tube (ET) with intermittent bag mask ventilation. Artificial tears were placed on eyes and the animal prepped and draped in sterile fashion. The ferret was placed in the supine position, and a vertical, approximately 3 cm midline incision was made through the skin of the anterior neck, centered about the cricothyroid membrane. The strap muscles were separated at the midline raphe and the anterior larynx and trachea were identified. A vertical midline incision was made through the cricoid cartilage and first two tracheal rings. An elliptical defect in the airway was created by removing the medial aspect of the cricoid and trachea on each side of the incision, resulting in a defect approximately 3 × 7 mm. The graft was cut to approximately ∼6–8 mm × 12 mm and secured with 5–0 prolene or PDS sutures (Ethicon, Raritan, NJ, USA) to secure the graft over the cricotracheal defect. Tisseel fibrin glue (Baxter International, Deerfield, Illinois, USA) was then applied over the graft according to the manufacturer's instruction to create an air-tight seal. The strap muscles were reapproximated with 3-0 vicryl sutures (Demetech, Miami, FL, USA) and the skin layer closed with 4-0 vicryl sutures (Demetech) followed by application of skin adhesive (3 M Vetbond; Saint Paul, Minnesota, USA). Antisedan (0.4 mg kg^−1^, intramuscular) was administered at the end of the case for reversal of xylazine. The animal was monitored for 4–5 weeks post-operatively prior to euthanasia and necropsy.

**Table 2:** Characterization of *in vivo* laryngotracheal reconstruction graft implantation

Animal no.	Airway % increase
1	1.40
2	9.77
3	9.69
4	11.61
Avg	8.12 ± 4.57

#### Endoscopic imaging

To investigate defect healing and mucosalization of the inner tracheal lumen following LTR, endoscopic imaging was obtained at 2- and 4-weeks post-operation. This was performed with a 0° 2.7 mm rod endoscope with ClaraMed phone endoscope adapter (São Paulo, Brazil) for photodocumentation using the Videoscope phone app (Amazipoint technology Ltd, Taiwan). Images were qualitatively evaluated for the presence of mucosalization, stenosis, granulation tissue, and scarring.

### Computed tomography (CT) imaging

Computed tomography (CT) imaging (Siemens Somatom Force, Munich, Germany) was acquired pre-operatively and at the time of euthanasia following LTR to evaluate airway diameter and graft integration. 3D slicer (https://www.slicer.org) was used to analyze the images in DICOM format. Segmentation of the laryngeal cartilage was performed along with separate segmentations of the presumed graft and airway. The volume of the airway segment was measured in the axial view from the superior cricoid to the first second tracheal ring keeping the number of CT slices consistent across images. The percent increase was calculated. As the ferret had a normal airway to start the experiment and a defect in the airway was created and then reconstructed with the graft, maintenance of at least the same diameter compared to baseline would be considered a successful reconstruction.

#### Histological analysis of *in vivo* implanted grafts

The cricotracheal specimen at the level of the graft implantation was harvested immediately following euthanasia. The tissue was then fixed in 10% NBF and embedded in low melt paraffin to prepare 5 μm sections which were mounted on glass slides and stained with H&E and SafO with Fast green counterstain to evaluate the degree of neocartilage formation, as described above. The samples were imaged with light microscopy.

### Statistical analysis

Statistical analysis was performed using Prism (GraphPad Software, San Diego, CA, USA). Quantitative data are presented as the mean ± standard deviation. Statistical analysis for biochemical data was performed using unpaired *t*-test and one-way ANOVA with *post-hoc* Sidak. Cell viability analysis was performed with unpaired *t*-test and Young's modulus with one-way ANOVA with *post-hoc* Sidak. Paired *t*-test was performed for airway volume statistics. Statistically significant values are presented as * *p* < 0.05, ** *p* < 0.01, ***p < 0.001, and **** *p* < 0.0001.

## Results

### MSC phenotyping

#### Cell isolation and CFU potential

Mononuclear cells were successfully isolated from each of the 3 ferret donor femora and identified in primary expansion as plastic-adherent, spindle-shaped cells, consistent with MSC morphology^45^ (ESI Fig. 2A†). MSCs were first identified from bone marrow mononuclear cells as fibroblastic colony-forming units (CFU-Fs).^46^ The CFU assay provides a measure of stemness within a given cell population as well as downstream differentiation potential,^47^ although it may not provide insight into *in vivo* cell behavior.^48^ All 3 donor fMSCs exhibited some degree of CFU potential (ESI Fig. 2B and C†), which was greater when cells were seeded at 100 cells per cm^2^ compared to 50 cells per cm^2^ in all donors with the exception of donor 2.

#### Chondrogenic differentiation of fMSCs in aggregate culture

**Fig. 1:**
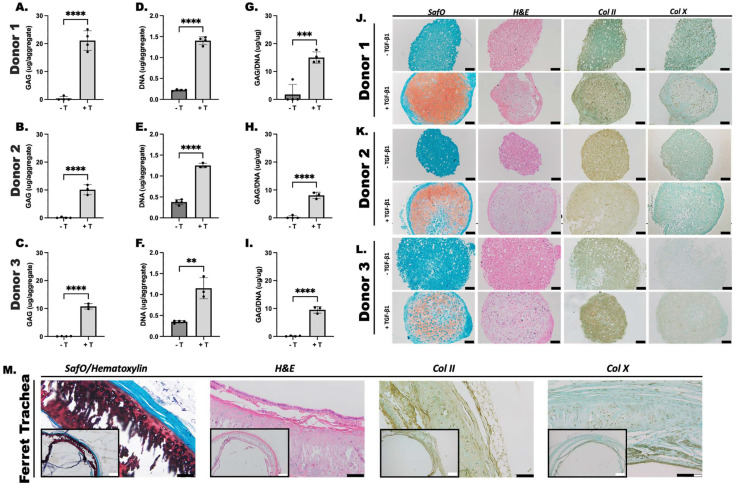
Chondrogenic differentiation of ferret mesenchymal stem cell (fMSC) aggregates. Glycosaminoglycan (GAG) (A–C), DNA (D–F), and GAG/DNA content (G–I) of fMSC aggregates from three donors was assessed after 3 weeks *in vitro* culture with (+T) and without (−T) TGFβ-1 supplementation. Histologic characterization of Donor 1 (J), 2 (K) and 3 (L) fMSC aggregates with and without (±) TGFβ-1 supplementation after 3 weeks of *in vitro* culture. Tissue sections were stained with Safranin O (SafO) for GAG (pink/red), hematoxylin and eosin (H&E), collagen II (col II), and collagen X (col X). Ferret trachea tissue staining is provided for reference (M). Scale bars, white = 500 μm; black = 100 μm. **** *p* < 0.0001.

Cell aggregates were cultured in pro-chondrogenic medium with or without supplementation with TGFβ-1 to examine the capacity of the fMSC aggregates to undergo directed chondrogenic differentiation following culture for 3 weeks. GAGs are the primary component of cartilage ECM.^49^ Thus, GAG content was measured as an indicator of cartilage formation (Fig. 1A–C). The fMSC aggregates cultured in the presence of TGFβ-1 produced significantly greater GAG relative to control, TGFβ-1-free cultures across all 3 donors. Additionally, DNA content of aggregates was analyzed to assess cell viability and proliferation (Fig. 1D–F). Average DNA content of TGFβ-1 treated cells at the culture endpoint was greater than 0.625 μg, the total theoretical amount present in the 2.5 × 10^5^ cells used to form each aggregate (assuming ∼2.5 pg of DNA per nucleus^50^). GAG/DNA production in cultures supplemented with GF compared to GF-free cultures in all donors followed a similar trend to results obtained for GAG content (Fig. 1G–I).

Histological sections were stained with SafO to indicate the presence of sulfated GAG (sGAG), a predominant marker of cartilage ECM (Fig. 1J–L). IHC staining was performed to assess the distribution of the predominant collagen of hyaline cartilage,^40^ col II, within the cellular aggregates. SaFO staining and tissue morphologically resembling cartilage appears throughout the central regions of the TGFβ-1-differentiated aggregates in all donors which is not observed in control aggregates. The histology results correspond to the biochemical data for all donors, with the most intense orange/red stain (SafO) observed in donor 1, which also had increased GAG content compared to donors 2 and 3. H&E staining further demonstrated chondrocyte morphology in TGFβ-1-differentiated aggregates. Subjective assessment reveals more intense and homogenous col II staining in chondrogenic differentiated aggregates compared to controls in donors 1 and 3 (Fig. 1J and L). Donor 2 aggregates showed staining in both control and GF-treated aggregates (Fig. 1K). Col X staining was performed to assess for hypertrophic cartilage.^51^ Faint staining was apparent in donor 3 aggregates cultured with TGFβ-1 (Fig. 1L) and minimal staining in the remainder of the aggregates. Ferret tracheal staining (Fig. 1M) and knee staining (ESI Fig. 3†) is provided for reference.

#### Osteogenic differentiation of fMSCs in monolayer culture

**Fig. 2:**
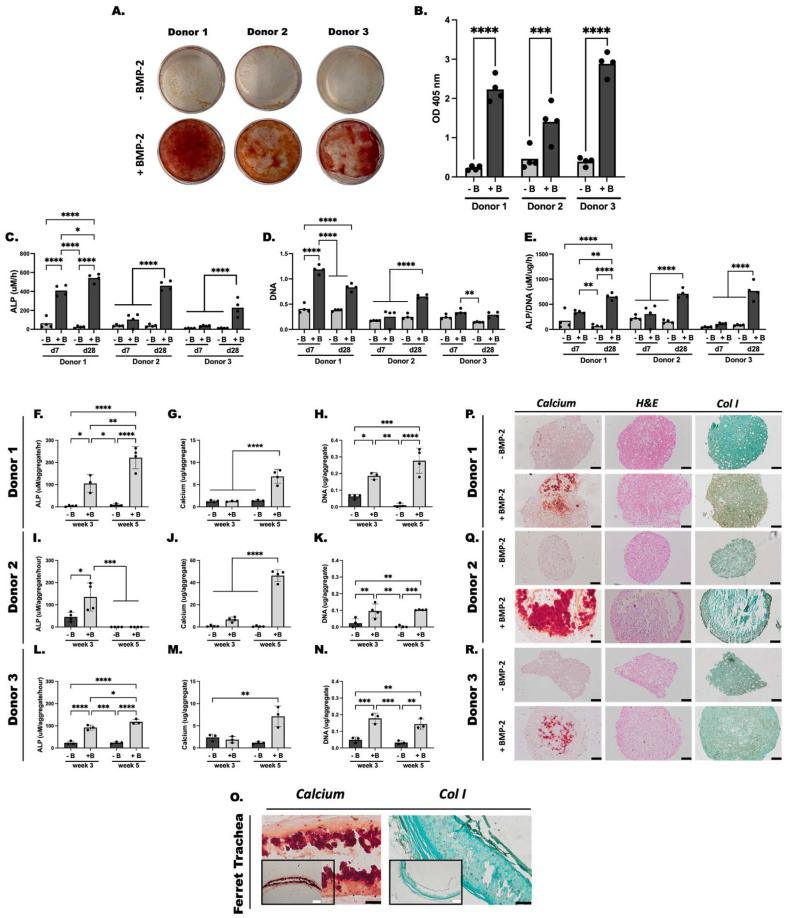
Osteogenic differentiation of ferret mesenchymal stem cells (fMSCs) in monolayer and aggregate culture. (A) Alizarin Red S (ARS) staining of monolayer fMSCs from three donors after 4 weeks *in vitro* culture in pro-osteogenic medium with (+B) and without (−B) BMP-2 is shown. (B) Semi-quantitation of ARS stain from monolayer samples. (C) Alkaline phosphatase (ALP) activity, (D) DNA content, and (E) ALP/DNA of monolayer fMSCs after 7 (d7) and 28 (d28) days of *in vitro* culture in pro-osteogenic medium. (F–L) ALP activity, (G–M) calcium content, and (H–N) DNA content of fMSC aggregates cultured *in vitro* in pro-osteogenic medium for 3 and 5 weeks with (+B) and without (−B) BMP-2. Histologic characterization of Donor 1 (P), 2 (Q) and 3 (R) fMSC aggregates with and without (±) BMP-2 supplementation after 5 weeks of *in vitro* culture. Tissue sections were stained with ARS for calcium, hematoxylin and eosin (H&E), and collagen type I (col I). Ferret trachea tissue staining is provided for reference (O). Scale bars, white = 500 μm; black = 100 μm. *** *p* < 0.001, **** *p* < 0.0001.

Osteoblasts produce osteoid matrix which becomes mineralized.^52^ Therefore, calcium content of the aggregates represents MSC osteogenic differentiation and bone formation. ARS staining was performed to visually stain calcium deposition and for semi-quantification of extracted ARS. All donor fMSCs underwent osteogenic differentiation when cultured with BMP-2 compared to GF-free controls (Fig. 2A) as evidenced by robust ARS staining. Semi-quantification of ARS extraction showed significantly greater ARS extraction in the BMP-2 treatment groups when compared to controls (Fig. 2B).

ALP activity was then measured as it is an early marker of osteogenesis^53^ (Fig. 2C). Supplementation of all donors with BMP-2 resulted in significantly higher ALP activity at day 28 compared to controls. Donor 1 also showed significantly elevated ALP at day 7 compared to controls while no differences were observed between conditions in the remaining donors at this time point. DNA content of the cells was also analyzed to assess cell viability and proliferation during osteogenic differentiation (Fig. 2D). DNA content of donor 1 at day 7 and 28 in BMP-2 treated cells was significantly elevated compared to BMP-2-free cells at their respective timepoints. DNA of donor 2 BMP-2 treated cells was significantly elevated compared to BMP-2 free cells at day 28 only, whereas DNA content of donor 3 at day 7 and 28 showed no difference between the two groups. ALP normalized to DNA content (ALP/DNA) showed a similar pattern across donors with BMP-2 treated cells demonstrating significantly elevated ALP/DNA at day 28 compared to BMP-2 free cells at that timepoint (Fig. 2E).

#### Osteogenic differentiation of fMSCs in aggregate culture

Osteogenic differentiation of fMSCs in aggregate culture with and without BMP-2 treatment was assessed after 3 and 5 weeks of culture. Biochemical and histologic results demonstrate GF-mediated osteogenesis of fMSCs. Results demonstrate elevated ALP activity in aggregates cultured with BMP-2 at weeks 3 and 5 compared to control aggregates in both donor 1 and 3 cells (Fig. 2F and H). Donor 2 on the other hand showed significantly elevated ALP activity only at week 3 in the BMP-2 cultured aggregates compared to BMP-2 free aggregates (Fig. 2G). Calcium content was also assessed as a marker of osteogenic differentiation (Fig. 2I–K). Calcium content of cell aggregates cultured with BMP-2 was significantly elevated by week 5 compared to all other conditions and timepoints in donors 1 and 2 (Fig. 2I and J). Donor 3 aggregates cultured with BMP-2 demonstrated significant calcium content compared to BMP-2 free aggregates at week 3 (Fig. 2K). DNA content of aggregates cultured with BMP-2 was significantly elevated at weeks 3 and 5 compared to BMP-2 free aggregates, although no significant difference was found between week 3 and 5 in all donors (Fig. 2L–N).

Histologic staining was used to examine the presence of markers of bone formation in BMP-2 and BMP-2-free cultures after 5 weeks of culture (Fig. 2O–Q). Of note, BMP-2-free aggregates were small and did not easily lend themselves to sectioning. When cultured with BMP-2, aggregates stained for ARS at week 5 in all donors which was not observed in BMP-2-free cultures. Two of four donor 3 aggregates cultured with BMP-2 did not stain for ARS. IHC was also performed to assess for col I, the predominant collagen found in bone and a hallmark of fibrocartilage and fibrotic scar.^39,40^ Donor 1 and 3 aggregates showed more homogenous col I stain throughout the aggregates cultured with BMP-2 whereas donor 2 aggregates stained at the periphery in the region that did not become mineralized. GF-free cultures also showed some faint staining for col I, notably donors 2 and 3. Ferret tracheal staining (Fig. 2R) and knee (ESI Fig. 3†) is provided for reference.

#### Effect of cell density on chondrogenic differentiation in 3D bioprinted discs

The effect of cell density on chondrogenic differentiation in 3D bioprinted cylindrical discs was assessed (ESI Fig. 4†). The 10E6 and 20E6 groups showed significantly increased GAG production compared to the 5E6 and control group. DNA was significantly elevated in the 10E6 and 20E6 groups compared to 5E6 and controls, but no notable was difference between 10 and 20E6 was demonstrated.

### Implant analysis

#### Preparation and characterization of 3D bioprinted scaffolds

**Fig. 3:**
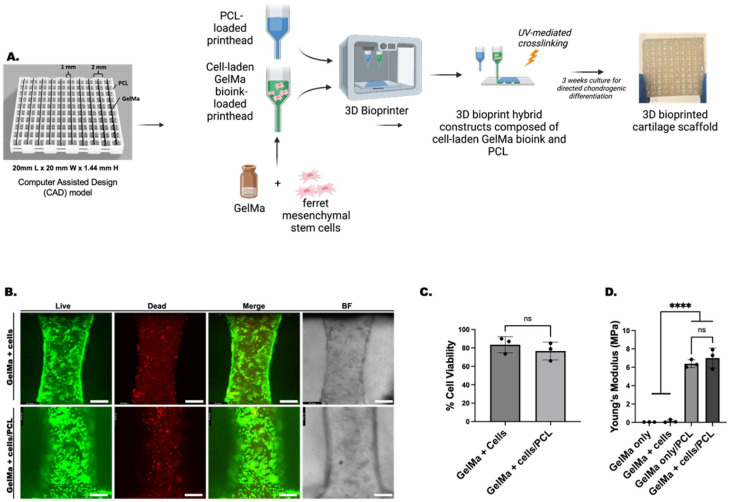
3D bioprinting process and scaffold characterization. (A) Schematic showing the 3D bioprinting process of GelMa + cells/PCL scaffolds. (B) Live (green)/dead (red) viability assay of 3D bioprinted scaffolds at day 1 post-print. (C) Cell viability comparison of GelMa + cells and GelMa + cells/PCL scaffolds at day 1 post-print. (D) Mechanical testing of scaffolds after 3 weeks *in vitro* culture in pro-chondrogenic medium supplemented with TGFβ-1. BF = bright field; ns = not significant. GelMa = gelatin methacrylate; PCL = polycaprolactone. Scale bar, white = 231.8 μm. **** *p* < 0.0001.

Fig. 3A and ESI Fig. 5† demonstrates the bioprinting process used to engineer hybrid scaffolds composed of GelMa bioink with incorporated fMSCs co-printed with PCL used in all subsequent studies. Live/dead analysis of the 3D bioprinted scaffolds was evaluated to determine the effect of the bioprinting process and co-printing with PCL on cell viability (Fig. 3B and C). No significant differences in cell viability were observed day 1 post-print between GelMa + cells and GelMa + cells/PCL scaffolds. GelMa + cells demonstrated 83.5 ± 8.7% cell viability compared to GelMa + cells/PCL which showed 76.6 ± 9.7% viability, indicating that co-printing with PCL did not significantly impact cell viability compared to printing bioink alone. Moreover, cell distribution of stained live/dead cells revealed homogenously incorporated cells within the GelMa bioink filament.

Compression testing of the scaffolds revealed an average Young's modulus of 7.92 ± 1.10 MPa for GelMa + cells/PCL scaffolds, 6.39 ± 0.43 MPa for GelMa only/PCL scaffolds, 0.31 ± 0.16 MPa for GelMa + cells scaffolds, and 0.05 ± 0.01 MPa for GelMa only scaffolds (Fig. 3D). The hybrid scaffolds, regardless of the presence of cells, demonstrated significantly increased Young's modulus compared to GelMa only and GelMa + cells scaffolds.

#### Chondrogenic differentiation of *in vitro* 3D bioprinted scaffolds

**Fig. 4:**
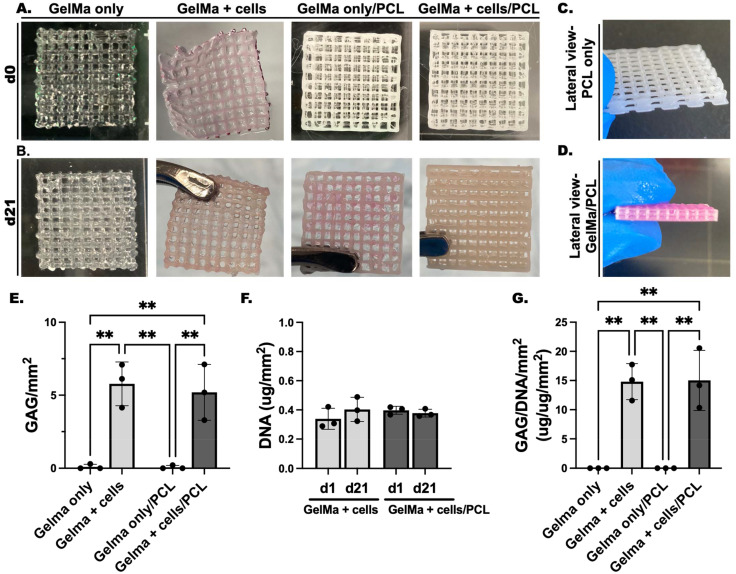
Chondrogenic differentiation of 3D bioprinted scaffolds. Gross images of (A) GelMa only, GelMa + cells, GelMa only/PCL, and GelMa + cells/PCL scaffolds at (A) day 0 and (B) day 21 of *in vitro* culture in pro-chondrogenic medium supplemented with TGFβ-1 reveals increased opacity of cell-laden bioink during culture. (C) Lateral view of PCL only scaffolds and (D) GelMa only/PCL scaffolds at day 1 demonstrates distinct layers within the scaffold. (E) GAG (μg) per mm^2^, (F) DNA (g) per mm^2^, and (G) GAG/DNA per mm^2^ content was assessed after 3 weeks of *in vitro* culture. GelMa = gelatin methacrylate; PCL = polycaprolactone. ** *p* < 0.01.

Chondrogenic differentiation in 3D bioprinted scaffolds was evaluated following 3 weeks of culture in pro-chondrogenic medium supplemented with TGFβ-1 (Fig. 4). Cell-incorporated scaffolds at this timepoint demonstrate increased opacity compared to both day 0 scaffolds and cell-free scaffolds, likely due to cartilage ECM production (Fig. 4A and B). Furthermore, GelMa + cells scaffolds were more easily handled following the culture period, allowing for increased ease of manipulation relative to GelMa only scaffolds. Fig. 4C and D demonstrates the distinct layers of PCL within the scaffold.

#### Biochemical analysis of *in vitro* scaffolds

Biochemical analysis of GAG, DNA, and GAG/DNA content relative to area (mm^2^) was evaluated to assess chondrogenic differentiation of the GelMa + cells and GelMa + cells/PCL scaffolds relative to controls (Fig. 4E–G). Quantitative results show significantly increased GAG per mm^2^ in the cell-incorporated scaffolds (Fig. 4E), and no difference was observed between GelMa + cells and GelMa + cells/PCL scaffolds which showed an average of 5.78 ± 1.50 and 5.20 ± 1.91 GAG (μg) per mm^2^, respectively. No effect of co-printing with PCL on chondrogenic differentiation was observed. Additionally, DNA per mm^2^ content immediately after printing, on day 1, and after 3 weeks of culture was evaluated to assess cell survival during the culture period (Fig. 4F). GelMa + cells and GelMa + cells/PCL scaffolds both showed no significant difference between DNA content at day 1 and day 21. Cell-free scaffolds did not reveal any measurable DNA content (data not shown). Both GelMa + cells and GelMa + cells/PCL scaffolds demonstrated similar and stable DNA content during the culture period (Fig. 4G) suggesting that cells are differentiating rather than proliferating.^54^

#### Histologic and immunohistochemical analysis of *in vitro* scaffolds

**Fig. 5:**
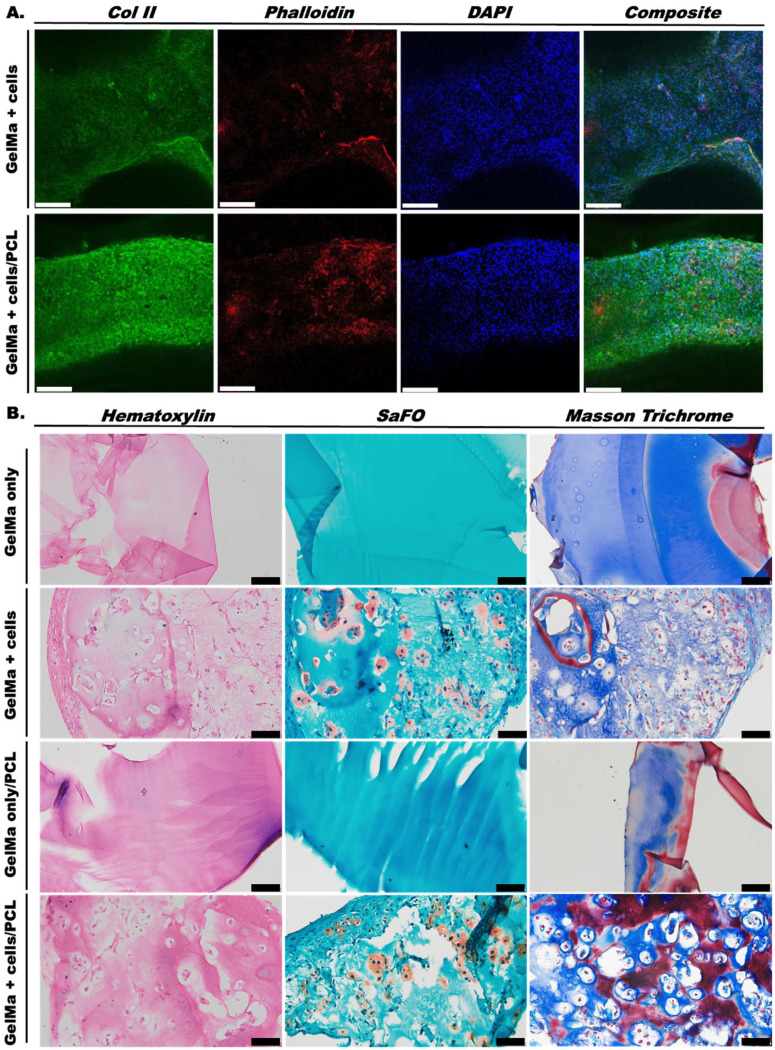
Histologic assessment of 3D bioprinted scaffolds. (A) Immunohistochemical analysis was performed on scaffolds after 3 weeks of culture in pro-chondrogenic medium. Scaffolds were assessed for col II, phalloidin, and DAPI with composite overlay presented. (B) Paraffin-embedded tissue samples were stained with hematoxylin, SafO, and Masson Trichrome. GelMa = gelatin methacrylate; PCL = polycaprolactone; Col II = collagen type II; SafO = Safranin O. Scale bars, white = 231.8 μm; black = 100 μm.

Col II IHC was performed to establish hyaline cartilage formation (Fig. 5A). Col II deposition appears pericellular within both GelMa + cells and GelMa + cells/PCL scaffolds at day 21 and qualitative assessment indicates increased col II in GelMa + cells/PCL scaffolds compared to GelMa + cells scaffolds. Phalloidin, a marker of cytoskeletal protein F-actin, was used to evaluate the cell interaction with the surrounding bioink and cell morphology. No significant differences between the cell-incorporated scaffolds were observed. DAPI staining of cell nuclei shows homogenous distribution of cells in the 3D bioprinted filaments after 3 weeks of culture. Composite images of col II, phalloidin, and DAPI further demonstrate the pericellular nature of col II staining, indicating cell-mediated production of ECM.

Histologic analysis *via* SafO, Masson trichrome, and H&E staining assessed for GAG content and cartilage morphology (Fig. 5B). Results indicate that GelMa + cells and GelMa + cells/PCL scaffolds undergo chondrogenesis as evidenced by the presence of chondrocyte morphology and SafO staining for sGAGs. Masson trichrome further demonstrates chondrocyte morphology and cell distribution. However, because gelatin, the base polymer used to prepare GelMa, is composed of soluble proteins prepared by partial hydrolysis of native collagen,^55^ there is evidence of collagen staining (blue) within the controls and no significant differences are observed between groups.

#### *In vivo* findings

**Fig. 6:**
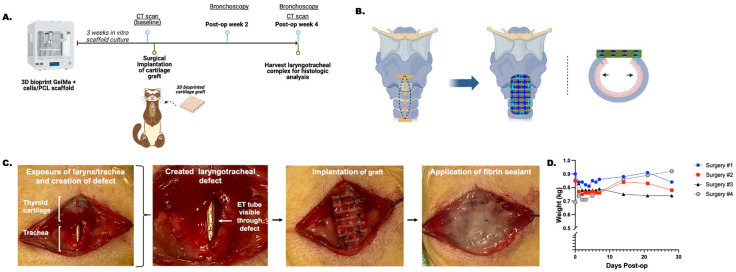
*In vivo* implantation of 3D bioprinted graft in a ferret model of laryngotracheal reconstruction. (A) Timeline of *in vivo* study. (B) Schematic diagram of LTR with graft implantation at the level of the inferior thyroid cartilage and proximal tracheal rings. (C) Gross images of the surgical steps to perform LTR. (D) LTR was performed on 4 ferrets and the weights tracked over the course of one-month post-operation. LTR = laryngotracheal reconstruction. ET = endotracheal tube.

A summary of the ferrets is provided in Table 2. A schematic of an average timeline of *in vivo* experiments is presented Fig. 6A along with a schematic of the surgery (Fig. 6B), and gross images of the surgical steps (Fig. 6C). Immediately post-operatively the ferrets experienced mild weight loss that did not exceed 10% of their pre-operative body weight (Fig. 6D). No animals died during the surgical procedure. Animal no.3 developed a skin ulcer post-operatively secondary to buprenorphine necrosis,^56^ and animal no. 4 experienced emesis and diarrhea with bright red blood per rectum immediately post-operatively and was treated with oral metronidazole 15 mg kg^−1^ twice daily for 5 days for possible colitis. No additional complications including surgical site infection, crepitus, stridor, or labored respiration was observed in the ferrets post-operatively. No surgical site anastomotic issues or dehiscence occurred.

#### Endoscopy and CT imaging

**Fig. 7:**
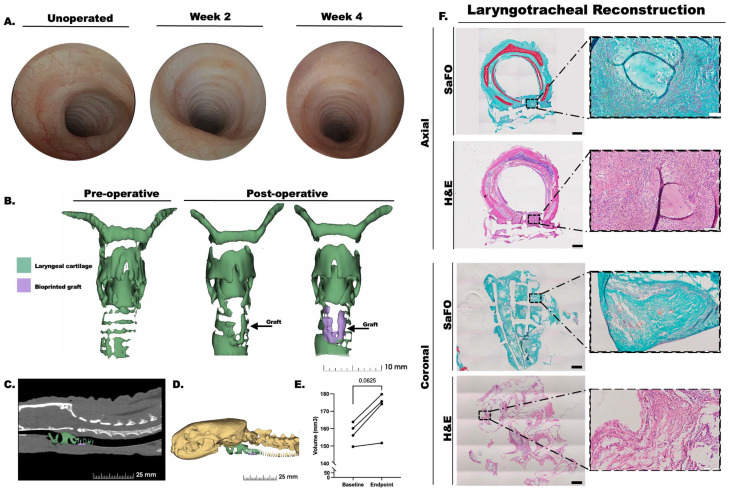
Ferrets as a biomedical research model for laryngotracheal reconstruction (LTR) (*n* = 4). (A) Bronchoscopy images were obtained at 2- and 4-weeks post-graft implantation in a ferret model of LTR revealing defect healing and mucosalization of the tracheal lumen at the site of implantation. (B) CT images of the implant site were used to generate 3D reconstructed models of the airway before and at the study endpoint (4 weeks). CT images demonstrate location of the graft (purple) within the laryngeal cartilages (green) in a sagittal section (C) and on 3D reconstructed sagittal view (D). (E) CT images of the implant site were used to obtain volume measurements of the airway before surgery (baseline) and at the study endpoint (4 weeks) which shows increased airway volume post-operatively. (F) *In vivo* graft histology shows Safranin O (SafO) and hematoxylin and eosin (H&E) stain following 1-month post-operation LTR implantation in the ferret. Tracheas were sectioned in the axial and coronal planes. Scale bars, black = 1 mm; white = 100.

Endoscopy revealed healing of the surgical defect along with smooth luminal surface of the subglottis and proximal trachea as well as mucosalization of the inner lumen in all the ferrets at weeks 2 and 4 post-operation Fig. 7A. No airway stenosis, granulation tissue, or subglottic collapse was noted in any of the ferrets at either timepoint, and CT imaging further verified the absence of granulation tissue or stenosis at the endpoint of the study (Fig. 7B and C). The airway diameter significantly increased post-operatively by 8.12% ± 4.57 (Fig. 7D, and Table 2).

#### *In vivo* histologic analysis

After 4 weeks, histologic examination of the tissue present between PCL strands showed fibrous tissue with some areas of SafO and presence of chondrocyte morphology in LTR histology samples, however overall cartilage architecture was minimal (Fig. 7F). Similar results were observed in the subcutaneously implanted grafts (ESI Fig. 6†). Of note, the graft proved difficult to section and PCL filaments resulted in vacant sites within the sectioned tissue.

## Discussion

With the advancement of personalized therapies to replace injured or diseased tissues, innovative stem cell-based therapeutics have progressed along with the fields of 3D bioprinting and regenerative medicine. In the current study, fMSC-laden cartilage grafts were 3D bioprinted for application in a ferret model of LTR. FMSCs, not previously reported in the literature, capable of directed musculoskeletal differentiation were first generated, providing the initial step towards surgical implantation of fMSC-populated grafts. A multi-material, 3D bioprinted hybrid scaffold composed of fMSC-laden GelMa co-printed with PCL capable of *in vitro* cartilage formation was then designed and fabricated. Evaluation of the engineered cartilage graft in a ferret model of LTR demonstrated healing of the defect site with epithelialization of the inner lumen mucosa and expansion of the airway despite minimal cartilage present at the graft site after 4 weeks. The overall purpose of this present study was to provide a proof-of-principle platform to demonstrate the therapeutic potential of 3D bioprinted fMSC derivatives in a ferret animal model of airway reconstruction.

The capacity to fully evaluate the conditions essential to the success of cellular therapy is not possible in humans. Progression toward a cellular therapy for LTR will require a preclinical animal model that closely resembles the physiology and function of the human. The ferret is an ideal animal model to test the therapeutic potential of novel regenerative therapies in the airway.^23,24^ In fact, recent work demonstrates the generation of ferret induced pluripotent stem cells (iPSCs) and compares the gene signature profile of ferret iPSCs to those of human, porcine, and mouse iPSCs.^24^ Results demonstrate that the ferret iPSC transcriptomic profile is most similar to primed human iPSCs, highlighting the similarity between humans and ferrets and driving further interest in deriving ferret stem cells.

The current study is the first to successfully isolate fMSCs and demonstrate the musculoskeletal differentiation potential of these cells. MSCs are defined by the International Society for Cellular Therapy by: (1) adherence on tissue culture plastic; (2) *in vitro* differentiation capacity into adipocytes, chondrocytes, and osteoblasts; and (3) presence of specific cell surface markers. Here, the plastic-adherent cells (ESI Fig. 1†) isolated from ferret femora are shown to undergo musculoskeletal differentiation towards chondrocytes and osteoblasts. The GF-mediated differentiation potential of these cells was investigated in a high cell density aggregate system which can closely recapitulate native developmental and healing processes and has application in many tissue types including cartilage^34,37^ and bone.^36,38^ TGFβ-1 mediated chondrogenic differentiation resulted in ∼84-fold increase in GAG production after 21 days of culture compared to controls across 3 donors (Fig. 1A–C). Additionally, monolayer fMSCs and high cell density aggregates (Fig. 2) cultured in pro-osteogenic medium demonstrated BMP-2-mediated osteogenic differentiation as measured by ALP and calcium analysis, of which aggregates showed significant increase compared to controls (Fig. 2F–K). These results demonstrate the multipotential differentiation capacity of the isolated cells. Of note, MSC donor-to-donor variability in differentiation capacity documented in the literature prompted investigation of 3 donor populations in this work which may necessitate the use of an ‘off-the-shelf’ printed construct to bypass donor variability using pre-screened cells (Fig. 1 and 2).^36,57–59^ Additionally, although fMSCs capable of musculoskeletal differentiation were generated in this work, further studies are warranted to further characterize the surface markers of these cells given that reports suggest that the same panel of surface antigens expressed in human MSCs may not be expressed across different species.^60,61^

The generation of fMSCs provides the first step toward fabrication of fMSC-derived cartilage for implantation in a pre-clinical ferret research model. 3D bioprinting allows for precise placement of live cells to spatially control tissue formation.^13^ Here, multi-material scaffolds were 3D bioprinted to generate tissue with properties derived from both chosen biomaterials (Fig. 3A, ESI Fig. 5†), GelMa and PCL. GelMA, a hydrogel produced through the chemical reaction of the gelatin backbone with methacrylic anhydride, a photocrosslinkable moiety,^42^ was used as a cell carrier.^13,42,62,63^ Due to the thermo-reversible properties of GelMa,^64^ it can be successfully printed without the need for supportive molds and immediately photo-crosslinked in order to maintain the filament structure.^65^ Ideally, encapsulated cells within the hydrogel transition towards cartilage tissue formation, secreting ECM that then replaces the hydrogel as it degrades.^63^ Conversely, PCL is an FDA-approved biodegradable, biocompatible thermoplastic polymer devoid of cell adhesion properties;^66–68^ it degrades *via* hydrolysis over the course of ∼1.5–2 years.^63,69^ PCL is used here as a reinforcement material to engineer scaffolds with suitable mechanical integrity to stent and expand the airway until the surrounding tissue fully matures. Co-printing cell-laden GelMa bioink filaments with PCL filaments has shown promising application in musculoskeletal regeneration.^64,70–72^ To the best of our knowledge, this work is the first to describe the layer-by-layer 3D bioprinting of MSC-laden GelMa co-printed with PCL without a sacrificial material or supportive mold capable of neocartilage formation. Future efforts will aim to fabricate constructs with more complex shapes, an achievement currently limited by challenges in resolution quality of existing extrusion-based 3D bioprinters.^13^

The fabrication of multi-material structures with precise design is an important strategy made uniquely possible by 3D bioprinting strategies. Additionally, Kang *et al.*^63^ reports that generation of porous constructs, like that used in this work, could potentially overcome the diffusion limit of 100–200 μm (ref. 63) necessary for cell survival and bypass challenges associated with scale-up when transitioning to clinical trials. As noted, the mechanical properties of the engineered tissue are an important factor as the tissue must possess sufficient strength to withstand surgical manipulation and avoid collapse of the expanded trachea. Here, the bioprinted graft ideally provided a rigid framework for the cartilaginous airway while simultaneously allowing for the influx of host cells within the porous spaces of the construct design. The composite scaffolds, regardless of the presence of cells, showed ∼6–7-fold increase in Young's modulus compared to that of GelMa only scaffolds (Fig. 3D). The Young's Modulus of native cartilage has been reported between 0.85 and 7.9 MPa,^73^ the upper limit of which is comparable to that of the composite scaffolds.

Formation of cartilage-like tissue was achieved in the *in vitro* cultured, cell-laden scaffolds as determined by GAG content (Fig. 4E–G), histologic evidence of col II by IHC (Fig. 5A) and SafO staining and presence of chondrocyte morphology (Fig. 5B). The secretion of cartilage-specific ECM in the composite constructs was similar to those in the cell-laden GelMA constructs, suggesting that co-printing with PCL did not interfere with fMSC differentiation. During cultivation, DNA content remained stable between day 1 and day 21 of culture in both the GelMa + cells and GelMa + cells/PCL scaffolds (Fig. 4F), likely secondary to differentiation of fMSCs towards chondrocytes which tend to senesce with maturation and stop proliferating.^74^ Moreover, increased opacity of cell-laden GelMa filaments visualized during the culture process of cell-incorporated scaffolds (Fig. 4A), indicates ECM production which was accompanied by increased handling capability of the GelMa + cells compared to GelMa alone scaffolds.

With regard to implantation, few studies using bioprinted constructs have assessed therapeutic efficacy of engineered grafts at orthotopic sites^13^ and similarly few studies have evaluated tissue engineering strategies for LTR.^14,43,44,75–78^ Although promising results in LTR have been achieved using conventional 3D printing strategies,^14,78^ these strategies lack extensive characterization of the implanted scaffolds. Additionally, despite the benefits of the ferret as a biomedical research model in airway physiology, the current study is the first to investigate airway reconstruction in ferrets. Potential complications of LTR, including restenosis due to the generation of scar and granulation tissue, submucosal fibrosis, or graft extrusion did not occur here.^79^ Mucosalization of the inner lumen was observed by week 2 post-implantation (Fig. 7A) after which point potential for stenosis or granulation tissue overgrowth decreases due to the presence of a confluent epithelial lining.^9^ It has been suggested that porous grafts, such as that used here, are more readily incorporated by the host and thus suitable for tracheal reconstruction.^9^ The graft was able to successfully stent and dilate the anterior cricoid and tracheal cartilage, resulting in an ∼8% increase in airway volume which approaches significance (Fig. 7E; Table 2). Additionally, it is important to note that at 4-week follow-up, no adverse impact on airway patency or structure was observed and there was no evidence of subcutaneous emphysema, the latter of which would suggest lack of healing at the defect site.

Despite successful surgical outcomes, minimal neo-cartilage was evident at the graft site at 1-month post-operation. Although there is evidence that donor cells can be found several weeks after implantation in both immunocompromised and immunocompetent animal models,^80^ little is known regarding what proportion of cells tend to survive implantation. The minimal cartilage phenotype present on histology suggests degradation of GelMa and lack of structural support for *in vitro* differentiated chondrocytes to maintain their phenotype *in vivo versus* possible chondrolysis and cell death following implantation. With regard to the latter, the need for sustained bioactive factor presentation (*e.g.*, TGFβ-1) to maintain a differentiated state may be necessary,^31,81^ which would require development of constructs capable of sustained bioactive factor delivery.^34,36^ Over the long term, particularly if performing longer tracheal segment reconstruction, lack of neocartilage formation could potentially predispose to airway abnormalities and collapse but may not be a relevant issue for LTR, as demonstrated here, given the successful surgical outcomes. Future work will aim to determine the fate of implanted cells and further investigate the optimal conditions necessary to maintain fMSC-derived cartilage with longer-term analyses. Additionally, both male and female donor MSCs will need to be investigated in future studies along with further investigation into cell surface markers of these cells and gene expression profiles. Although further studies are warranted, this work presents the possibility of successful reconstruction of the cricoid and trachea by means of 3D bioprinting.

## Conclusion

This work presents the first report describing the application of fMSCs for musculoskeletal regeneration. Additionally, a 3D bioprinting strategy employing the layer-by-layer bioprinting of PCL and MSC-laden GelMa scaffolds to create engineered cartilage-like tissue was applied in a ferret airway surgical model. Using a CAD design and commercially available 3D bioprinter, rapid construct formation on the centimeter scale was achieved. The ability to generate multi-material, porous constructs that allow for mass transport and diffusion of oxygen and nutrients bypasses challenges associated with scale up when transitioning to larger animal models or clinical trials. The printed constructs can be easily harvested and manipulated, and the system can also accommodate the delivery of multiple cell types (*e.g.*, mature differentiated cells, embryonic stem cells, induced pluripotent stem cells) and bioactive molecules (*e.g.*, growth factors, gene delivery) within the bioink to address basic biological questions and generate engineered tissue. Despite promising surgical outcomes following implantation, minimal cartilage tissue was evident at the study endpoint. In summary, we have established the generation of fMSCs for regenerative applications and the use of the ferret in airway reconstruction, which have not previously been described. Future work to further optimize stem cell-based, 3D bioprinting strategies will enhance the development of engineered cartilage for therapeutic intervention in airway pathology.

## Conflicts of interest

There are no conflicts of interest to declare.

## Supplementary Material

BM-013-D4BM01251H-s001

## Data Availability

All data will be made available upon reasonable request.
