# Supplementary material for: 3D bioprinted ferret mesenchymal stem cell-laden cartilage grafts for laryngotracheal reconstruction in a ferret surgical model
Source: Biomater Sci. 2025 Jan 22;13(5):1304–22. doi: 10.1039/d4bm01251h (PMC11784027; doi:10.1039/d4bm01251h)
Supplement: BM-013-D4BM01251H-s001 [file BM-013-D4BM01251H-s001.pdf]

Supplementary Information (SI) for Biomaterials Science.
This journal is © The Royal Society of Chemistry 2025

Supplementary Material:

3D Bioprinted Ferret Mesenchymal Stem Cell-Laden Cartilage Grafts for Laryngotracheal Reconstruction in a Ferret Surgical Model

Alexandra McMillan[1, 2], Matthew R. Hoffman[1], Yan Xu[2], Zongliang Wu[2], Adreann Peel[3], Allan Guymon[3], Sohit Kanotra[1], Aliasger K. Salem*

[1]Department of Otolaryngology, University of Iowa Hospitals and Clinics, Iowa City, IA
[2]Department of Pharmaceutical Sciences and Experimental Therapeutics, College of Pharmacy, University of Iowa, Iowa City, IA
[3]Department of Chemical and Biochemical Engineering, University of Iowa, Iowa City, IA

* Corresponding author

This document is provided as supplemental material to the manuscript titled "3D Bioprinted Ferret Mesenchymal Stem Cell Scaffolds for Laryngotracheal Reconstruction in a Ferret Surgical Model".

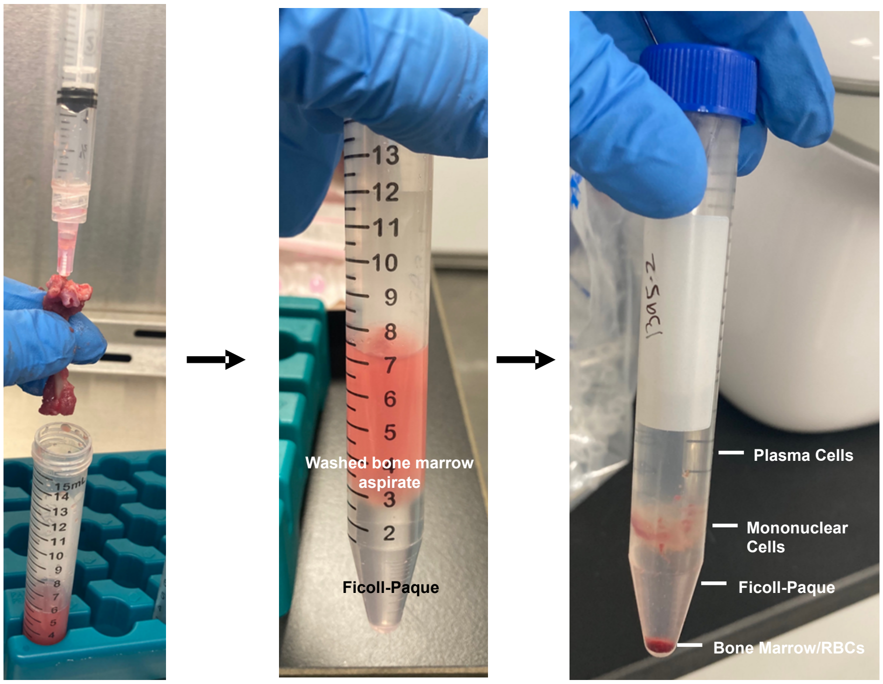

Supplemental Figure 1. Initial cell isolation. FMSCs were isolated from ferret femora via density gradient centrifugation with Ficoll-Paque and harvested from the mononuclear cell layer for further expansion on tissue culture plates. FMSCs = ferret mesenchymal stem cells.

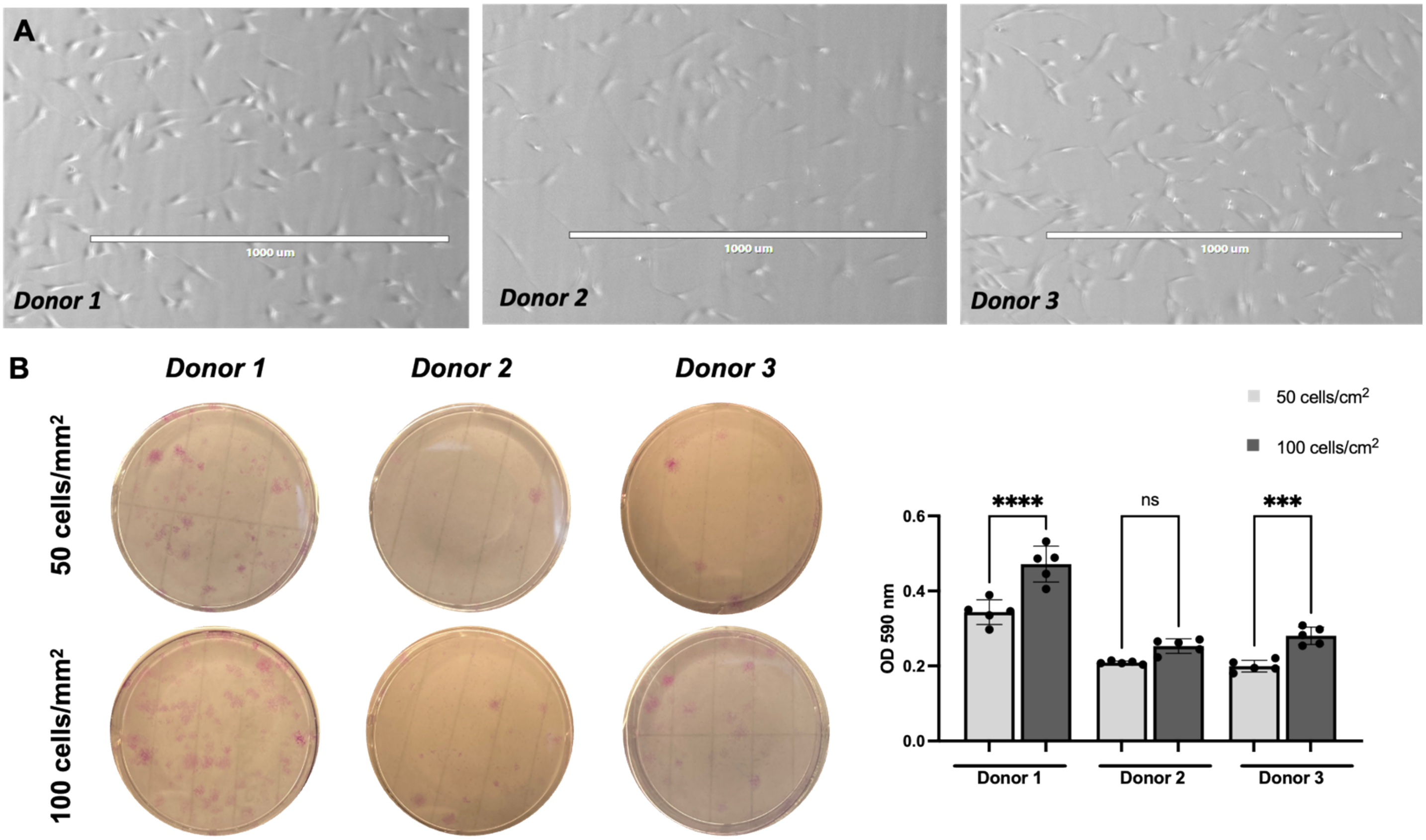

Supplemental Figure 2. Initial fMSC culture and colony forming unit (CFU) potential. A) Passage 1 fMSCs from 3 ferret donors were isolated and expanded on tissue culture plastic, demonstrating spindle-shaped cells, consistent with MSC morphology after 4 days of culture. **B)** FMSCs from 3 ferret donors were plated at clonal density to assess CFU potential of the cell population. Plates were stained with 0.3% crystal violet after 7 days of culture followed by semi-quantification of crystal violet stain with acetic acid. FMSCs = ferret mesenchymal stem cells.

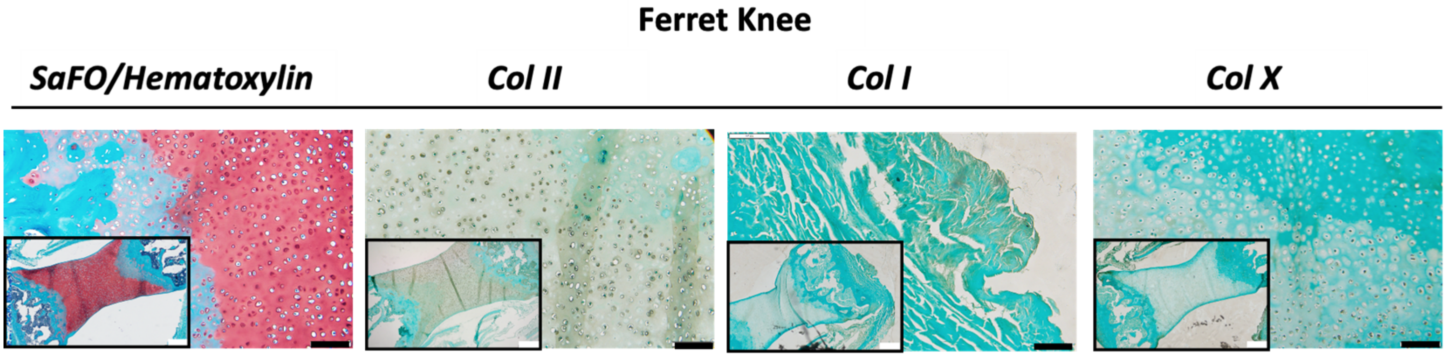

Supplemental Figure 3. Ferret knee histologic staining. Tissue sections were stained with Safranin O for GAG (pink/red), hematoxylin, collagen II, collagen I, and collagen X. Ferret trachea tissue staining is provided for reference. SafO = Safranin O; col II- collagen type II; col I = collagen type I; col X - collagen type X.

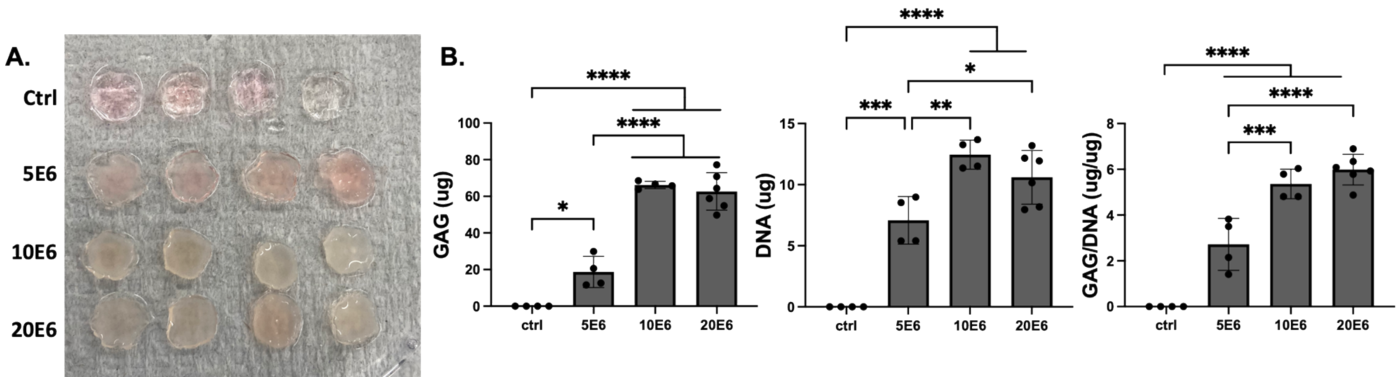

Supplemental Figure 4. 3D bioprinted GelMa + cells discs with varying cell density. A) Gross images of discs with no cells (Ctrl), 5E6, 10E6, and 20E6 cells/ml GelMa after 3 weeks of culture in pro-chondrogenic medium supplemented with TGFß-1. **B)** Biochemical analysis of discs assessed GAG, DNA, and GAG/DNA per disc. * $p< 0.05$, ** $p< 0.01$, *** $p<0.001$, and **** $p < 0.0001$.

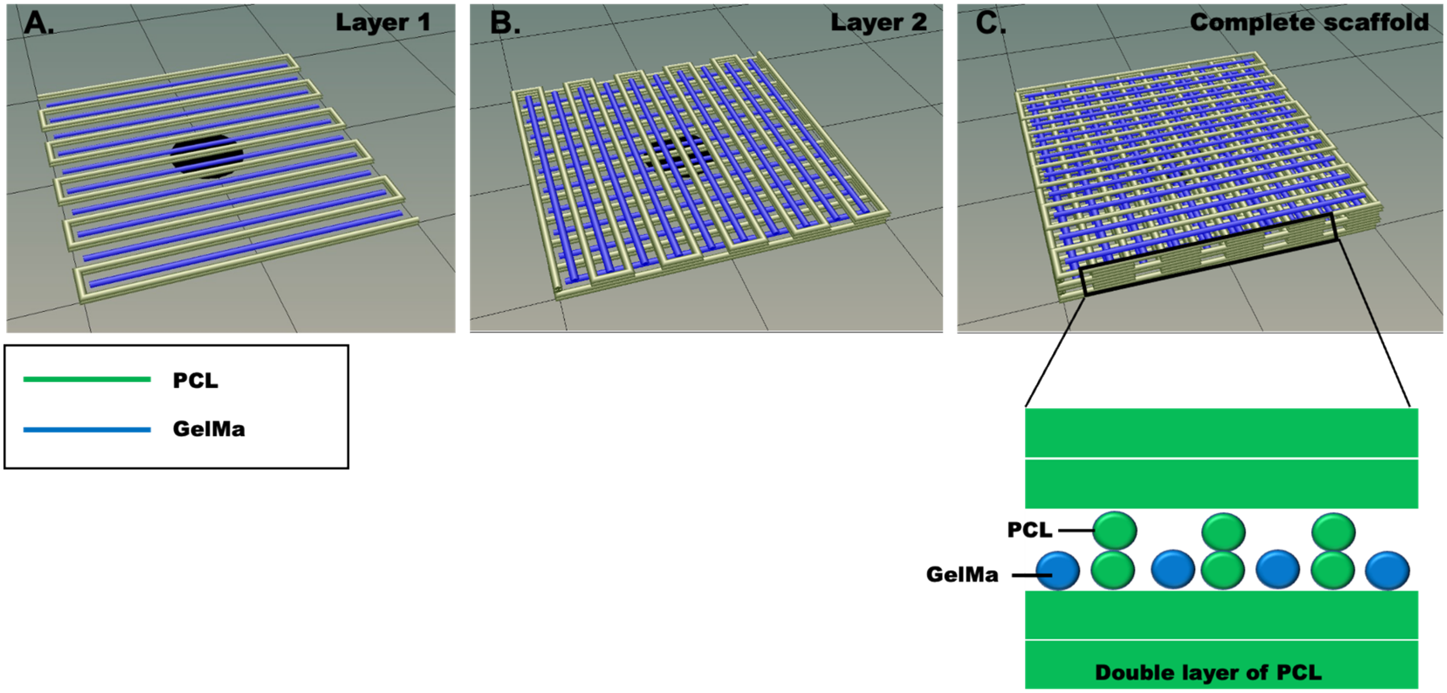

Supplemental Figure 5. Schematic demonstrating the process of 3D bioprinting used to generate scaffolds composed of GelMa, with or without cells, co-printed with PCL. One layer of GelMa was printed for every two repeated layers of PCL except for the final top layer which was composed of single layer of each. **A)** The first of five layers with consecutive paths printed in an alternating 0-90° perpendicular pattern was fabricated by printing a double layer of PCL with GelMa bioink then printed between the PCL strands. **B)** The second layer was printed at a 90° perpendicular pattern in a similar fashion to layer 1. C) The completed scaffold is shown with zoomed inset to further demonstrate that one layer of GelMa was printed for every two repeated, stacked filaments of PCL.

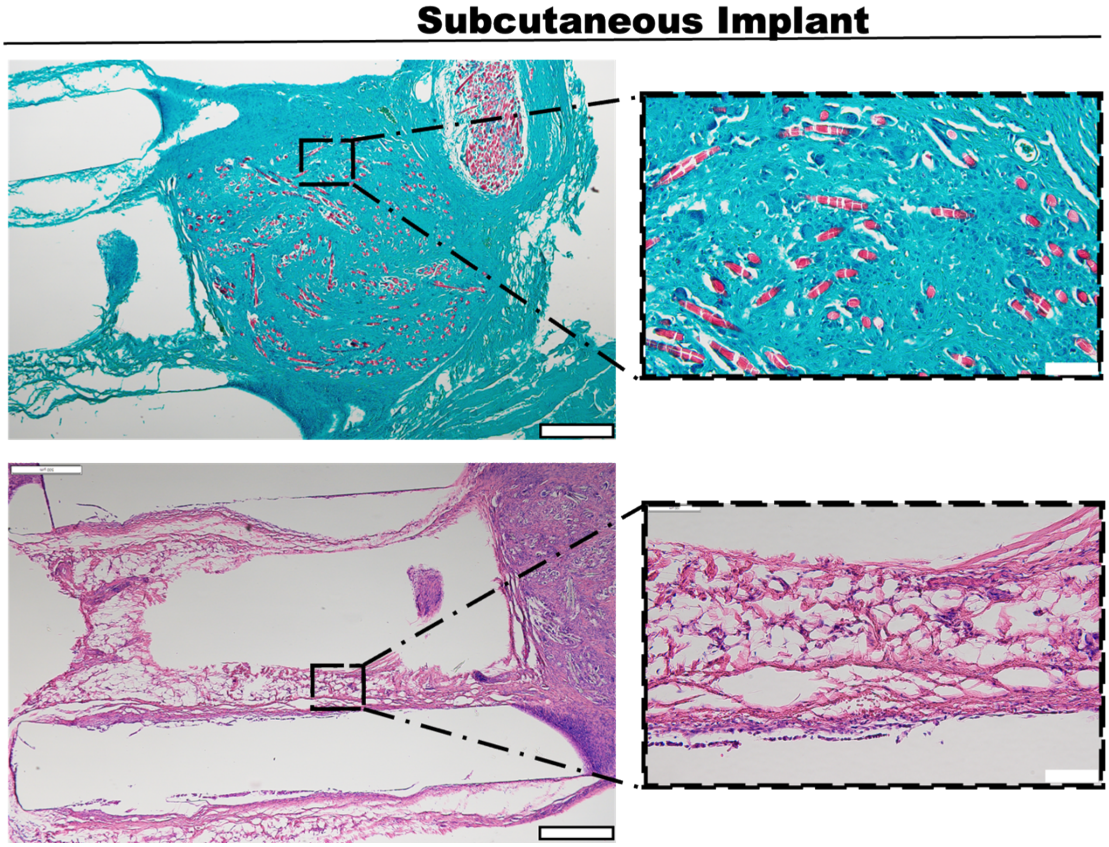

Supplemental Figure 6. In vivo subcutaneous graft implantation. In vivo graft histology of subcutaneous implanted grafts in a ferret. Safranin O (SaFO) and hematoxylin and eosin (H&E) stains were performed on the tissue harvested at the 5-week post-operation timepoint. Scale bars, white = 100; hollow= 500 µm.
